# Role of cardiovascular magnetic resonance in the guidelines of the European Society of Cardiology

**DOI:** 10.1186/s12968-016-0225-6

**Published:** 2016-01-22

**Authors:** Florian von Knobelsdorff-Brenkenhoff, Jeanette Schulz-Menger

**Affiliations:** Working Group Cardiovascular Magnetic Resonance, Experimental and Clinical Research Center, a joint cooperation between the Charité Medical Faculty and the Max-Delbrueck Center for Molecular Medicine; and HELIOS Klinikum Berlin Buch, Department of Cardiology and Nephrology, Berlin, Germany

**Keywords:** Cardiovascular magnetic resonance, Guideline, Cardiology, Reimbursement

## Abstract

**Background:**

Despite common enthusiasm for cardiovascular magnetic resonance (CMR), its application in Europe is quite diverse. Restrictions are attributed to a number of factors, like limited access, deficits in training, and incomplete reimbursement. Aim of this study is to perform a systematic summary of the representation of CMR in the guidelines of the European Society of Cardiology (ESC).

**Methods:**

Twenty-nine ESC guidelines were screened for the terms “magnetic”, “MRI”, “CMR”, “MR” and “imaging”. As 3 topics were published twice (endocarditis, pulmonary hypertension, NSTEMI), 26 guidelines were finally included. MRI in the context of non-cardiovascular examinations was not recognized. The main CMR-related conclusions and, if available, the level of evidence and the class of recommendation were extracted.

**Results:**

Fourteen of the 26 guidelines (53.8 %) contain specific recommendations regarding the use of CMR. Nine guidelines (34.6 %) mention CMR in the text, and 3 (11.5 %) do not mention CMR. The 14 guidelines with recommendations regarding the use of CMR contain 39 class-I recommendations, 12 class-IIa recommendations, 10 class-IIb recommendations and 2 class-III recommendations. Most of the recommendations have evidence level C (41/63; 65.1 %), followed by level B (16/63; 25.4 %) and level A (6/63; 9.5 %). The four guidelines, which absolutely contained most recommendations for CMR, were stable coronary artery disease (*n* = 14), aortic diseases (*n* = 9), HCM (*n* = 7) and myocardial revascularization (*n* = 7).

**Conclusions:**

CMR is represented in the majority of the ESC guidelines. They contain many recommendations in favour of the use of CMR in specific scenarios. Issues regarding access, training and reimbursement have to be solved to offer CMR to patients in accordance with the ESC guidelines.

## Background

Cardiovascular magnetic resonance (CMR) has been applied in a wide variety of indications in clinical cardiology. The most frequent indications are inflammatory and ischemic heart disease as well as cardiomyopathies. But also in rare diseases like amyloidosis, as well as in congenital heart disease, CMR has demonstrated its usefulness [1, 2]. CMR provides detailed information about cardiovascular anatomy and function by combining diverse techniques. In particular, the characterization of the myocardial tissue including the detection of oedema and the highly resolved determination of fibrosis is a unique feature of CMR [3]. Furthermore, with myocardial stress-perfusion imaging – free of ionizing radiation and with high diagnostic accuracy – the large patient group with (suspected) coronary artery disease is addressed [4]. Finally, the introduction of robust and fast imaging techniques as well as targeted examination protocols facilitated the clinical use [5].

Despite common enthusiasm for this modality, its use in Europe is quite diverse. This restriction is attributed to a number of factors, like missing skills both to run a CMR examination and to interpret the images under integration of profound cardiologic knowledge; relatively high costs and incomplete reimbursement; and limited access to scanners with cardiac dedication.

Aim of this study is to perform a systematic summary of the representation of CMR in the guidelines of the European Society of Cardiology (ESC) in order to stimulate the discussion about future plans for training, distribution and reimbursement of CMR in Europe.

## Methods

All ESC guidelines, which are listed on the ESC website (http://www.escardio.org/Guidelines-&-Education/Clinical-Practice-Guidelines/ESC-Clinical-Practice-Guidelines-list/listing) were collected (Table 1). If more than one guideline for the same topic has been published in this period, both were analysed for changes, but only the most recent was included in the final analysis. The documents were screened for the terms “magnetic”, “MRI”, “CMR”, “MR” and “imaging”. MRI in the context of non-cardiovascular examinations like brain MRI was not recognized. The main conclusions were extracted and if available, the level of evidence and the class of recommendation were given (Tables 2 and 3). The number in parenthesis behind the citation provides the page of the fulltext guideline. If a recommendation refers to “imaging” in general, it was registered if the context included CMR. This process was done twice for every guideline to reassure that no relevant information was missed. The absolute number of recommendations is finally summarized, whereby equal recommendations that appeared in more than one guideline were only counted once. The order of the guidelines is chronologic beginning with the most recent. Guidelines other than by the ESC as well as ESC position statements were not included to guarantee one common level of guideline.

**Table 1:** List of ESC guidelines used for this summary. 1 = guideline contains specific recommendations regarding the use of CMR; 2 = guideline mentions scenarios in which CMR may be used, but without giving any specific recommendation; 3 = guideline does not mention CMR at all

Nr.	Title	Year	Role of CMR
1	ESC Guidelines for the management of patients with ventricular arrhythmias and the prevention of sudden cardiac death [6]	2015	1
2	ESC/ERS Guidelines for the diagnosis and treatment of pulmonary hypertension [7]	2015	2
3	ESC guidelines for the management of acute coronary syndromes in patients presenting without persistent ST-segment elevation [9]	2015	1
4	ESC Guidelines for the diagnosis and management of pericardial diseases [11]	2015	1
5	ESC Guidelines for the management of infective endocarditis [12]	2015	1
6	ESC Guidelines on diagnosis and management of hypertrophic cardiomyopathy [14]	2014	1
7	ESC Guidelines on the diagnosis and treatment of aortic diseases [15]	2014	1
8	ESC/EACTS Guidelines on myocardial revascularization [16]	2014	1
9	ESC Guidelines on the diagnosis and management of acute pulmonary embolism [17]	2014	1
10	ESC/ESA Guidelines on non-cardiac surgery: cardiovascular assessment and management [18]	2014	1
11	ESC Guidelines on diabetes, pre-diabetes, and cardiovascular diseases developed in collaboration with the EASD [19]	2013	2
12	ESC guidelines on the management of stable coronary artery disease [20]	2013	1
13	ESC Guidelines on cardiac pacing and cardiac resynchronization therapy [21]	2013	2
14	ESH/ESC Guidelines for the management of arterial hypertension [22]	2013	1
15	ESC/EACTS Guidelines on the management of valvular heart disease [23]	2012	2
16	Focused update of the ESC Guidelines for the management of atrial fibrillation [24]	2012	3
17	ESC/ACCF/AHA/WHF Third universal definition of myocardial infarction [25]	2012	2
18	ESC Guidelines for the management of acute myocardial infarction in patients presenting with ST-segment elevation [26]	2012	1
19	ESC Guidelines for the diagnosis and treatment of acute and chronic heart failure [27]	2012	1
20	European Guidelines on cardiovascular disease prevention in clinical practice [28]	2012	2
21	ESC/EAS Guidelines for the management of dyslipidaemias [29]	2011	3
22	ESC Guidelines for the management of acute coronary syndromes in patients presenting without persistent ST-segment elevation [10]	2011	(new guideline 2015)
23	ESC Guidelines on the management of cardiovascular diseases during pregnancy [30]	2011	1
24	ESC Guidelines on the diagnosis and treatment of peripheral artery diseases [31]	2011	1
25	ESC Guidelines for the management of grown-up congenital heart disease [32]	2010	2
26	Focused Update of ESC Guidelines on device therapy in heart failure [34]	2010	3
27	Guidelines on the prevention, diagnosis, and treatment of infective endocarditis [23]	2009	(new guideline 2015)
28	Guidelines for the diagnosis and management of syncope [35]	2009	2
29	Guidelines for the diagnosis and treatment of pulmonary hypertension [8]	2009	(new guideline 2015)

**Table 2:** Class of recommendations

Class of recommendation	Definition	Suggested wording to use
Class I	Evidence and/or general agreement that a given treatment or procedure is beneficial, useful, effective.	Is recommended/is indicated
Class II	Conflicting evidence and/or a divergence of opinion about the usefulness/efficacy of the given treatment or procedure.	
Class IIa	Weight of evidence/opinion is in favour of usefulness/efficacy.	Should be considered
Class IIb	Usefulness/efficacy is less well established by evidence/opinion.	May be considered
Class III	Evidence or general agreement that the given treatment or procedure is not useful/effective, and in some cases may be harmful.	Is not recommended

**Table 3:** Level of evidence

Level of evidence A	Data derived from multiple randomized clinical trials or meta-analyses.
Level of evidence B	Data derived from a single randomized clinical trial or large non-randomized studies.
Level of evidence C	Consensus of opinion of the experts and/or small studies, retrospective studies, registries.

## Results

**Fig. 1:**
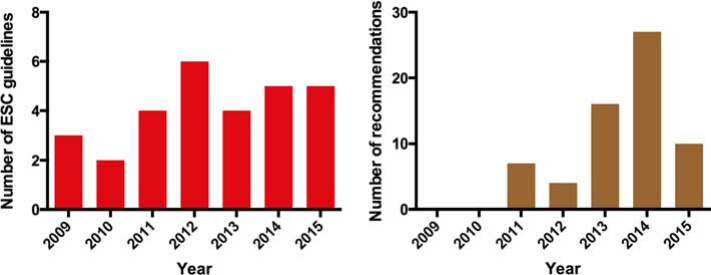
*Left:* Number of ESC guidelines screened for this analysis per year. *Right:* Number of specific recommendations regarding CMR per year

**Fig. 2:**
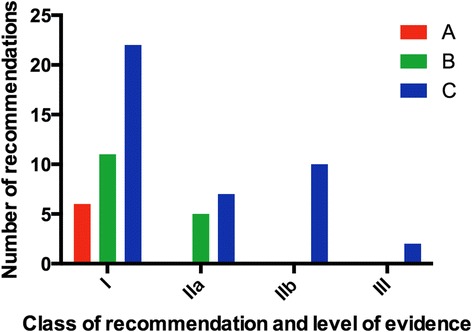
Class and level of the recommendations for CMR in the ESC guidelines

Of the 29 ESC guidelines we screened, three topics were covered twice (endocarditis 2015 and 2009, pulmonary hypertension 2015 and 2009, NSTEMI 2015 and 2011). Of the remaining 26 ESC guidelines, 14 (53.8 %) contain specific recommendations regarding the use of CMR (Fig. 1, Table 1). Nine guidelines (34.6 %; endocarditis, pulmonary hypertension, diabetes, pacing, heart valve disease, definition of infarction, prevention, congenital heart disease, syncope) principally mention scenarios in which CMR may be used, but without giving any specific recommendation. Three guidelines (11.5 %; atrial fibrillation, dyslipidaemias, device therapy in heart failure) do not mention CMR at all. The 14 guidelines with recommendations regarding the use of CMR contain 39 class-I recommendations, 12 class-IIa recommendations, 10 class-IIb recommendations and 2 class-III recommendations (Fig. 2). (The diverse recommendations for myocardial revascularization in dependency of the evidence of ischemia were only counted once as IA). Most of the recommendations have evidence level C (41/63; 65.1 %), followed by level B (16/63; 25.4 %) and level A (6/63; 9.5 %). The two class-III recommendations in the context of CMR are: i) In the guideline for pulmonary embolism, MR angiography should not be used to rule out pulmonary embolism. ii) In the guideline about assessment before non-cardiac surgery, imaging stress testing in general is not recommended before low-risk surgery. The four guidelines, which absolutely contained most recommendations with referral to CMR, were stable coronary artery disease from 2013 (*n* = 14), aortic diseases (*n* = 9), HCM (*n* = 7) as well as myocardial revascularization (*n* = 7) from 2014. Twenty-eight recommendations refer to stress-imaging, 17 recommendations refer to the vasculature, 7 to cardiomyopathies, 5 to left- and right-ventricular function assessment (in part including fibrosis imaging), 4 to the pericardium and 2 to myocarditis. A summary of clinical scenarios/diagnoses, where the ESC made recommendations regarding CMR, is provided in the appendix of this paper.

**Table 4:** Recommendations for CMR in patients with ventricular arrhythmias and for the prevention of sudden cardiac death

Non-invasive evaluation of patients with suspected or known ventricular arrhythmias	Class^a^	Level^b^	Page
Pharmacological stress testing plus imaging modality is recommended to detect silent ischaemia in patients with ventricular arrhythmias who have an intermediate probability of having coronary artery disease by age or symptoms and are physically unable to perform a symptom-limited exercise test.	I	B	12
CMR or CT should be considered in patients with ventricular arrhythmias when echocardiography does not provide accurate assessment of LV and RV function and/or evaluation of structural changes.	IIa	B	12
Management of ventricular arrhythmias in inflammatory heart disease	Class^a^	Level^b^	Page
Demonstration of persistent myocardial inflammatory infiltrates by immunohistological evidence and/or abnormal localized fibrosis by CMR after acute myocarditis may be considered as an additional indicator of increased risk of SCD in inflammatory heart disease.	IIb	C	53
Prevention of sudden cardiac death in athletes	Class^a^	Level^b^	Page
Upon identification of ECG abnormalities suggestive of structural heart disease, echocardiography and/or CMR imaging is recommended.	I	C	62

^a^ Class of recommendation

^b^ Level of evidence

### 2015 ESC guidelines for the management of patients with ventricular arrhythmias and the prevention of sudden cardiac death [6]

Table 4 summarizes the recommendations for CMR in the context of patients with ventricular arrhythmias and the prevention of sudden cardiac death.

**Table 5:** Recommendations for imaging in patients with suspected non-ST-elevation acute coronary syndromes

Recommendations for imaging in patients with suspected non-ST-elevation acute coronary syndromes	Class^a^	Level^b^	Page
In patients with no recurrence of chest pain, normal ECG findings and normal levels of cardiac troponin (preferably high-sensitivity), but suspected acute coronary syndrome, a non-invasive stress test (preferably with imaging) for inducible ischaemia is recommended before deciding on an invasive strategy.	I	A	15

^a^ Class of recommendation

^b^ Level of evidence

For family members of sudden unexplained death syndrome or sudden arrhythmic death syndrome victims, echocardiography and/or CMR is recommended (Appendix on page 11 of the guideline). In patients with sustained ventricular tachycardia or ventricular fibrillation, the recommended algorithm for further patient assessment includes CMR (Fig. 1 on page 14). For instance myocarditis should also be suspected and a CMR scan may reveal abnormal fibrotic myocardial tissue (page 54). In patients with non-ischaemic cardiomyopathy, CMR fibrosis imaging (using late gadolinium enhancement, LGE) is associated with increased risk of all-cause mortality, heart failure hospitalization and sudden cardiac death. The incremental value of LGE over other prognostic markers needs to be determined (page 36). Standardized evaluation of patients with HCM should include CMR in the case of inadequate echo window (page 39). LGE has been suggested to be used to guide ICD therapy in individuals with HCM with intermediate risk, however with few supportive data (page 38). Similarly, LGE on CMR of the right and left ventricle has been reported as risk factors for sudden cardiac death or appropriate ICD discharge in ARVC (page 40). In particular in ARVC, CMR provides excellent assessment of right ventricular size, function and regional wall motion. In paediatric patients with frequent premature ventricular complexes, cardiac evaluation including CMR is recommended (page 46). Non-invasive imaging of cardiac structure, best done by CMR, can be used to plan and guide ablation procedures for ventricular tachycardia (page 22).

### 2015 ESC/ERS guidelines for the diagnosis and treatment of pulmonary hypertension [7]

CMR is listed among the tests to contribute to the diagnosis of pulmonary hypertension, being accurate and reproducible in the assessment of right ventricular size, morphology and function and of blood flow, stroke volume, cardiac output, pulmonary arterial distensibility and right ventricular mass. The presence of LGE, reduced pulmonary arterial distensibility and retrograde flow have high predictive value for the identification of pulmonary hypertension. In patients with pulmonary hypertension, CMR may also be useful in cases of suspected congenital heart disease if echocardiography is not conclusive. MR angiography has a potential in patients with suspected chronic thromboembolic pulmonary hypertension. CMR provides useful prognostic information in patients with pulmonary artery hypertension (page 12). Specifically, right atrial size and the presence of pericardial effusion as assessed by CMR are used for risk assessment in pulmonary arterial hypertension (table 13 of the guideline). There is no specific recommendation regarding CMR and no significant change regarding the role of CMR in pulmonary hypertension between the present guideline and the 2009 version [8].

### 2015 ESC guidelines for the management of acute coronary syndromes in patients presenting without persistent ST-segment elevation [9]

CMR can assess both perfusion and wall motion abnormalities, and patients presenting with acute chest pain with a normal stress CMR have an excellent short- and midterm prognosis. CMR also permits detection of scar tissue and can differentiate this from recent infarction. CMR can facilitate the differential diagnosis between infarction and myocarditis or Tako-Tsubo cardiomyopathy (page 11). In subjects with no criteria for early invasive strategy, a non-invasive imaging stress test is recommended. No specific test is mentioned (Table 5). There is no significant change of the role of CMR compared to the 2011 NSTEMI guideline [10].

**Table 6:** Recommendations for CMR in pericardial diseases

Recommendation for diagnostic work-up of pericardial diseases	Class^a^	Level^b^	Page
CT and/or CMR are second-level testing for diagnostic workup in pericarditis	I	C	38
Recommendations for the diagnosis and management of pericarditis associated with myocarditis	Class^a^	Level^b^	Page
CMR is recommended for the confirmation of myocardial involvement	I	C	13
Recommendations for the diagnosis of pericardial effusion	Class^a^	Level^b^	Page
CT or CMR should be considered in suspected cases of loculated pericardial effusion, pericardial thickening and masses, as well as associated chest abnormalities	IIa	C	14
Recommendations for the diagnosis of constrictive pericarditis	Class^a^	Level^b^	Page
CT and/or CMR are indicated as second-level imaging techniques to assess calcifications (CT), pericardial thickness, degree and extension of pericardial involvement	I	C	17
Recommendations for therapy of constrictive pericarditis	Class^a^	Level^b^	Page
Empiric anti-inflammatory therapy may be considered in cases with transient or new diagnosis of constriction with concomitant evidence of pericardial inflammation (i.e. CRP elevation or pericardial enhancement on CT/CMR)	IIb	C	19

^a^ Class of recommendation

^b^ Level of evidence

### 2015 ESC guidelines for the diagnosis and management of pericardial diseases [11]

CMR has shifted towards a comprehensive imaging modality, allowing visualization and tissue characterization of the pericardium (and heart) in patients with pericardial disease and appraisal of the consequences of pericardial abnormalities on cardiac function and filling patterns (page 20). Table 12 of the guideline summarizes the contribution of different imaging modalities in various pericardial diseases and table 13 of the guideline compares non-invasive imaging modalities to study the pericardium. Thereby, CMR is predominantly ranked as good (“++”) or excellent (“+++”). Under the headline “what is new”, CMR is recommended for the detection of pericardial inflammation to identify forms of initial reversible constrictive pericarditis, allowing a trial of medical anti-inflammatory therapy (page 5). The evidence of pericardial inflammation by CMR is also mentioned as one diagnostic criterion for acute pericarditis (table 4 of the guideline). In patients with myocarditis, CMR is recommended for the confirmation of myocardial involvement (page 13). CMR may be helpful to detect loculated pericardial effusion and pericardial thickening and masses, as well as associated chest abnormalities. CMR can contribute to the differentiation of constrictive pericarditis and restrictive cardiomyopathy (table 10 of the guideline), e.g. by assessment of ventricular coupling with real-time cine magnetic resonance during free breathing (page 19). In some cases of pericardial cysts, CMR may be helpful (page 35). The recommendations made for CMR in pericardial diseases are summarized in Table 6.

**Table 7:** Recommendations for CMR in patients with HCM

Recommendations for CMR in patients with HCM	Class^a^	Level^b^	Page
It is recommended that CMR studies be performed and interpreted by teams experienced in cardiac imaging and in the evaluation of heart muscle disease	I	B	14
In the absence of contraindications, CMR with LGE is recommended in patients with suspected HCM who have inadequate echocardiographic windows, in order to confirm the diagnosis.	I	C	14
In the absence of contraindications, CMR with LGE should be considered in patients fulfilling diagnostic criteria for HCM, to assess cardiac anatomy, ventricular function, and the presence and extent of myocardial fibrosis.	IIa	B	14
CMR with LGE imaging should be considered in patients with suspected apical hypertrophy or aneurysm.	IIa	C	14
CMR with LGE imaging should be considered in patients with suspected cardiac amyloidosis.	IIa	C	14
CMR with LGE may be considered before septal alcohol ablation or myectomy, to assess the extent and distribution of hypertrophy and myocardial fibrosis.	IIb	C	14
CMR may be considered every 5 years in clinically stable patients, or every 2–3 years in patients with progressive disease.	IIb	C	37

^a^ Class of recommendation

^b^ Level of evidence

### 2015 ESC guidelines for the management of infective endocarditis [12]

Within the subchapter about ‘complications of infective endocarditis’ dealing with ‘myocarditis and pericarditis’, CMR is mentioned (next to echocardiography) to assess myocardial involvement during infective endocarditis (page 30). This CMR indication is new compared to the 2009 guideline [13].

### 2014 ESC guidelines on diagnosis and management of hypertrophic cardiomyopathy [14]

HCM in adults is defined by a wall thickness ≥15 mm and in first-degree relatives ≥13 mm in one or more LV myocardial segments - as measured by any imaging technique, including CMR (page 7, 8). Some patients with apical or distal hypertrophy develop small apical aneurysms, sometimes associated with myocardial scarring. These may only be detectable on CMR, ventriculography or contrast echo (page 9). The prevalence of non-sustained ventricular tachycardia increases with age and correlates with LV wall thickness and the presence of LGE on CMR (page 36). However, even though the extent of LGE on CMR has some utility in predicting cardiovascular mortality, current data do not support the use of LGE in prediction of sudden cardiac death risk (page 14). LGE at the right ventricular insertion points or localized to segments of maximum LV thickening on CMR assists for differentiating the diagnosis of hypertensive heart disease and HCM (table 9 of the guideline). The specific recommendations made for CMR in HCM are summarized in Table 7.

**Table 8:** Recommendations for CMR in aortic diseases

Recommendations on diagnostic work-up of acute aortic syndrome	Class^a^	Level^b^	Page
In stable patients with a suspicion of acute aortic syndrome, CMR is recommended (or should be considered) according to local availability and expertise	I	C	22
In case of initially negative imaging with persistence of suspicion of acute aortic syndrome, repetitive imaging (CT or CMR) is recommended.	I	C	22
In case of uncomplicated Type B aortic dissection treated medically, repeated imaging (CT or CMR) during the first days is recommended.	I	C	22
In uncomplicated Type B intramural hematoma, repetitive imaging (CMR or CT) is indicated.	I	C	26
In uncomplicated Type B penetrating aortic ulcer, repetitive imaging (CMR or CT) is indicated.	I	C	27
Recommendations for the management of aortic root dilation in patients with bicuspid aortic valve	Class^a^	Level^b^	Page
CMR or CT is indicated in patients with bicuspid aortic valve when the morphology of the aortic root and the ascending aorta cannot be accurately assessed by TTE.	I	C	42
In the case of aortic diameter >50 mm or an increase >3 mm/year measured by echocardiography, confirmation of the measurement is indicated, using another imaging modality (CT or CMR).	I	C	42
Recommendations for follow-up and management in chronic aortic diseases	Class^a^	Level^b^	Page
Contrast CT or CMR is recommended to confirm the diagnosis of chronic aortic dissection.	I	C	48
For follow-up after (T)EVAR in young patients, CMR should be preferred to CT for magnetic resonance-compatible stent grafts, to reduce radiation exposure.	IIa	C	48

^a^ Class of recommendation

^b^ Level of evidence

### 2014 ESC guidelines on the diagnosis and treatment of aortic diseases [15]

CMR is regarded as a valuable tool to image the aorta. On a scale from “+” to “+++”, the ease of use is graded as “++”, diagnostic reliability as “+++”, serial examinations as “+++”, and aortic wall visualization as “+++” (page 11). CMR is considered the leading technique for diagnosis of aortic dissection, with a reported sensitivity and specificity of 98 %. However, several methodological and practical limitations preclude the use of this modality in the majority of cases and in unstable patients (page 21). Recommendations for the use of CMR in patients with aortic diseases are given Table 8.

**Table 9:** Recommendations for CMR in the context of myocardial revascularization

Recommendations for imaging to determine ischemia to plan revascularization	Class^a^	Level^b^	Page
Stress CMR, stress-echo, SPECT or PET are recommended in subjects with intermediate pretest probability for suspected coronary artery disease and stable symptoms	I	A	14
To achieve a prognostic benefit by revascularization in patients with coronary artery disease, ischemia has to be documented by non-invasive imaging			
Left main disease with stenosis >50 %	I	A	18
Any proximal LAD stenosis >50 %	I	A	18
Two-vessel or three-vessel disease with stenosis > 50 % with impaired LV function (LVEF < 40 %)a	I	A	18
Large area of ischaemia (>10 % LV)	I	B	18
Single remaining patent coronary artery with stenosis >50 %	I	C	18
Recommendations for follow-up and management after myocardial revascularization for asymptomatic patients	Class^a^	Level^b^	Page
Early imaging testing should be considered in specific patient subsets.	IIa	C	72
Routine stress testing may be considered >2 years after PCI and >5 years after CABG.	IIa	B	72
Recommendations for follow-up and management after myocardial revascularization for symptomatic patients	Class^a^	Level^b^	Page
It is recommended to reinforce medical therapy and lifestyle changes in patients with low-risk findings at stress testing.	I	C	72
With intermediate- to high-risk findings at stress testing, coronary angiography is recommended.	I	C	72
Recommendation for carotid artery screening before CABG	Class^a^	Level^b^	Page
CMR, CT, or digital subtraction angiography may be considered if carotid artery stenosis by ultrasound is >70 % and myocardial revascularization is contemplated.	IIb	C	39

^a^ Class of recommendation

^b^ Level of evidence

### 2014 ESC/EACTS guidelines on myocardial revascularization [16]

This guideline contains recommendations for CMR both for determining myocardial ischemia and for follow-up patients after myocardial revascularization, as well as for preparation before surgical myocardial revascularization. Table 9 shows the specific recommendations. There is no clear recommendation for CMR viability testing. Even though CMR has a high diagnostic accuracy for assessing the transmural extent of myocardial scar tissue and contractile reserve, its ability to detect viability and predict recovery of wall motion is no better than other imaging techniques (page 15).

**Table 10:** Recommendation for CMR in pulmonary embolism

Recommendations for CMR in pulmonary embolism	Class^a^	Level^b^	Page
MR angiography should not be used to rule out pulmonary embolism.	III	C	11

^a^ Class of recommendation

^b^ Level of evidence

### 2014 ESC guidelines on the diagnosis and management of acute pulmonary embolism [17]

MR angiography, although promising, is not yet ready for clinical practice due to its low sensitivity, high proportion of inconclusive MR angiography scans, and low availability in most emergency settings (Table 10).

**Table 11:** Recommendations for CMR in the context of non-cardiac surgery

Recommendations for non-invasive stress testing of ischemic heart disease	Class^a^	Level^b^	Page
Imaging stress testing is recommended before high-risk surgery in patients with more than two clinical risk factors and poor functional capacity (<4 METs).	I	C	12
Imaging stress testing may be considered before high- or intermediate-risk surgery in patients with one or two clinical risk factors and poor functional capacity (<4 METs).	IIb	C	12
Imaging stress testing is not recommended before low-risk surgery, regardless of the patient’s clinical risk.	III	C	12

^a^ Class of recommendation

^b^ Level of evidence

### 2014 ESC/ESA guidelines on non-cardiac surgery: cardiovascular assessment and management [18]

Resting LV function can be evaluated before non-cardiac surgery in high-risk surgery (IIb, C). Following the guidelines, this can be done by radionuclide ventriculography, gated single photon emission computed tomography, echocardiography, CMR or multislice CT all with similar accuracy. The recommendations for non-invasive stress testing of ischemic heart disease are given in Table 11. As nuclear myocardial perfusion imaging and stress echocardiography were mainly used in clinical studies about preoperative ischemic testing, these modalities are pronounced. CMR (both perfusion and wall motion analysis) is mentioned as an accurate alternative method.

**Table 12:** Recommendations for CMR in stable coronary artery disease

Recommendations for non-invasive testing for ischemic heart disease	Class^a^	Level^b^	Page
In patients with suspected stable coronary artery disease and intermediate pretest probability of 15 % - 65 % and LVEF ≥50 %, stress imaging is preferred as the initial test option if local expertise and availability permit.	I	B	17
An imaging stress test is recommended as the initial test for diagnosing stable coronary artery disease if the pretest probability is between 66-85 % or if LVEF is <50 % in patients without typical angina.	I	B	17
An imaging stress test is recommended in patients with resting ECG abnormalities, which prevent accurate interpretation of ECG changes during stress.	I	B	17
An imaging stress test should be considered in symptomatic patients with prior revascularization (PCI or CABG).	IIa	B	17
An imaging stress test should be considered to assess the functional severity of intermediate lesions on coronary arteriography.	IIa	B	17
Recommendations for risk stratification using ischemia testing	Class^a^	Level^b^	Page
Risk stratification is recommended based on clinical assessment and the results of the stress test initially employed for making a diagnosis of stable coronary artery disease	I	B	22
Stress imaging for risk stratification is recommended in patients with a non-conclusive exercise ECG	I	B	22
Risk stratification using stress ECG (unless they cannot exercise or display ECG changes which make the ECG non evaluable) or preferably stress imaging if local expertise and availability permit is recommended in patients with stable coronary disease after a significant change in symptom level	I	B	22
Stress imaging is recommended for risk stratification in patients with known stable coronary artery disease and a deterioration in symptoms if the site and extent of ischemia would influence clinical decision making	I	B	22
In asymptomatic adults with diabetes or asymptomatic adults with a strong family history of coronary artery disease or when previous risk assessment testing suggests high risk of coronary artery disease, such as a coronary artery calcium score of 400 or greater stress imaging tests (MPI, stress echocardiography, perfusion CMR) may be considered for advanced cardiovascular risk assessment.	IIb	C	24
Recommendation for re-assessment in patients with stable coronary artery disease	Class^a^	Level^b^	Page
An exercise ECG or stress imaging if appropriate is recommended in the presence of recurrent or new symptoms once instability has been ruled out.	I	C	25
Reassessment of the prognosis using stress testing may be considered in asymptomatic patients after the expiration of the period for which the previous test was felt to be valid (“warranty period”)	IIb	C	25
In symptomatic patients with revascularized stable coronary artery disease, stress imaging (stress echocardiography, CMR or MPS) is indicated rather than stress ECG.	I	C	47
Late (6 months) stress imaging test after revascularization may be considered to detect patients with restenosis after stenting or graft occlusion irrespective of symptoms.	IIb	C	47

^a^ Class of recommendation

^b^ Level of evidence

### 2013 ESC guidelines on diabetes, pre-diabetes, and cardiovascular diseases developed in collaboration with the EASD [19]

Patients with glucose perturbations are in need of early risk assessment to identify co-morbidities and factors that increase cardiovascular risk. This includes among other the evaluation of myocardial viability and LV function by means of echo-Doppler and/or CMR (page 28), and Duplex ultrasonography, computed tomography angiography and CMR to evaluate carotid artery stenosis (page 45). To evaluate inducible ischaemia, only exercise testing, stress echocardiography, or myocardial scintigraphy are mentioned (page 28).

### 2013 ESC guidelines on the management of stable coronary artery disease [20]

Table 12 summarizes the corresponding recommendations for CMR in the context of stable coronary artery disease. CMR may be used to define structural cardiac abnormalities and evaluate ventricular function. Use of CMR is recommended in patients, in whom, despite the use of echo contrast agents, transthoracic echocardiography is unable to answer the clinical question (usually because of a restricted acoustic window) and who have no contra-indications for CMR (page 13). In patients with suspected coronary artery disease and intermediate pretest probability, non-invasive testing is recommended. Among the modalities to perform stress imaging, CMR is mentioned on the same level as stress echocardiography, SPECT and PET. To stratify the risk for events, high risk is assumed in stress CMR if there are ≥2/16 segments with new perfusion defects or ≥3 dobutamine-induced dysfunctional segments (page 20). CMR coronary arteriography must still be regarded primarily as a research tool and is not recommended for routine clinical practice in the diagnostic evaluation of suspected coronary artery disease (page 19).

**Table 13:** Recommendation for CMR in the management of arterial hypertension

Recommendations for stress-testing in arterial hypertension	Class^a^	Level^b^	Page
Whenever history suggests myocardial ischaemia, a stress ECG test is recommended, and, if positive or ambiguous, an imaging stress test (stress echocardiography, stress CMR or nuclear scintigraphy) is recommended.	I	C	21

^a^ Class of recommendation

^b^ Level of evidence

### 2013 ESC guidelines on cardiac pacing and cardiac resynchronization therapy [21]

Regarding patient selection for cardiac resynchronization therapy, it is mentioned that CMR and other imaging techniques were evaluated. However, the real value of these novel technologies remains to be determined in randomized trials (page 23). Furthermore, general safety-based recommendations for MR imaging in patients with implanted cardiac devices are given, according to conventional or MR-conditional devices (page 44).

### 2013 ESH/ESC guidelines for the management of arterial hypertension [22]

When searching for asymptomatic organ damage in patients with arterial hypertension, CMR should be considered for assessment of LV size and mass when echocardiography is technically not feasible and when LGE imaging would have therapeutic consequences (page 17). On a scale from “+” to “++++”, CMR was graded with “++” regarding cardiovascular predictive value, “+” regarding availability, “+++” regarding reproducibility” and “++” regarding cost-effectiveness (page 20). CMR is rated as highly sensitive to detect changes of LV hypertrophy, superior to echocardiography and ECG (page 51). Concerning renal artery stenosis as cause of secondary hypertension, CMR is named as additional/confirmatory test after renal ultrasonography (page 22). The recommendation for CMR in suspected ischemic heart disease in the context of hypertension-induced organ damage is given in Table 13.

**Table 14:** Recommendations for CMR in patients with STEMI

Recommendations for imaging during hospitalization and at discharge in patients with STEMI	Class^a^	Level^b^	Page
If echocardiography is not feasible, CMR may be used as an alternative for assessment of infarct size and resting LV function.	IIb	C	26
For patients with multivessel disease, or in whom revascularization of other vessels is considered, stress testing or imaging (e.g. using stress myocardial perfusion scintigraphy, stress echocardiography, positron emission tomography or CMR) for ischaemia and viability is indicated before or after discharge.	I	A	26

^a^ Class of recommendation

^b^ Level of evidence

### 2012 guidelines on the management of valvular heart disease [23]

In patients with inadequate echocardiographic quality or discrepant results, CMR should be used to assess the severity of valvular lesions - particularly regurgitant lesions - and to assess ventricular volumes and systolic function, as CMR assesses these parameters with higher reproducibility than echocardiography. CMR is the reference method for the evaluation of right ventricular volumes and function and is therefore useful to evaluate the consequences of tricuspid regurgitation. In practice, the routine use of CMR is limited because of its limited availability, compared with echocardiography (page 7).

In *aortic regurgitation*, CMR (or CT) is recommended for the evaluation of the aorta in patients with Marfan syndrome, or if an enlarged aorta is detected by echocardiography, particularly in patients with bicuspid aortic valves (page 10). Furthermore, CT or preferably CMR are advisable when the distal ascending aorta is not well visualized and/or when the surgical indication may be based on aortic enlargement, rather than LV size or function.

In *aortic stenosis* with paradoxical low flow, the diagnosis of severe AS requires careful exclusion of diverse reasons for the echo constellation before making the decision to intervene. In addition to more detailed echocardiographic measurements, this may require CMR and catheterization (page 14). (CT and) CMR provide additional information on the assessment of the ascending aorta when it is enlarged (page 14). Furthermore, CMR may also be useful for the detection and quantification of myocardial fibrosis, providing additional prognostic information in symptomatic patients without coronary artery disease (page 14).

In secondary mitral regurgitation and low LVEF, it is also mandatory to assess the absence, or presence and extent, of myocardial viability by one of the available imaging techniques (dobutamine echocardiography, SPECT, PET or CMR) (page 23). There are no specific recommendations for CMR in this guideline.

### 2012 focused update of the ESC Guidelines for the management of atrial fibrillation [24]

CMR is not mentioned in this guideline.

### 2012 third universal definition of myocardial infarction [25]

Imaging evidence of new loss of viable myocardium or new regional wall motion abnormality is listed among the criteria for acute myocardial infarction. Among the criteria for prior myocardial infarction, imaging evidence of a region of loss of viable myocardium that is thinned and fails to contract, in the absence of a non-ischaemic cause, is listed (page 3). CMR is mentioned next to other imaging tests (page 9) for assessing myocardial viability, perfusion, and function. Furthermore, its value in detecting myocardial disease states that can mimic myocardial infarct, such as myocarditis, is emphasized (page 10). There are no specific recommendations for CMR in this guideline.

### 2012 ESC guidelines for the management of acute myocardial infarction in patients presenting with ST-segment elevation [26]

Contrast-enhanced CMR is mentioned as one of several techniques to make the diagnosis of no-reflow. If, in spite of the angiography performed in the acute phase, there are concerns about inducible ischaemia, an outpatient exercise-testing or stress-imaging test (using scintigraphy, echocardiography or CMR) is appropriate. Regarding the assessment of viability, the same statement as within the revascularization guidelines from 2014 is given: CMR has a high diagnostic accuracy for assessing transmural extent of myocardial scar tissue, but its ability to detect viability and predict recovery of wall motion is not superior to other imaging techniques (page 27). Table 14 shows the recommendations for CMR in patients with STEMI.

**Table 15:** Recommendations for CMR in acute and chronic heart failure

Recommendations for CMR in ambulatory patients suspected of having heart failure	Class^a^	Level^b^	Page
CMR imaging is recommended to evaluate cardiac structure and function, to measure LVEF, and to characterize cardiac tissue, especially in subjects with inadequate echocardiographic images or where the echocardiographic findings are inconclusive or incomplete (but taking account of cautions/contraindications to CMR).	I	C	10
Myocardial perfusion/ischaemia imaging (echocardiography, CMR, SPECT, or PET) should be considered in patients thought to have coronary artery disease, and who are considered suitable for coronary revascularization, to determine whether there is reversible myocardial ischaemia and viable myocardium.	IIa	C	10

^a^ Class of recommendation

^b^ Level of evidence

### 2012 ESC guidelines for the diagnosis and treatment of acute and chronic heart failure [27]

CMR is particularly valuable in identifying inflammatory and infiltrative conditions as well as in the work-up of patients with suspected cardiomyopathy, arrhythmias, suspected cardiac tumours, or pericardial diseases, and is the imaging method of choice in patients with complex congenital heart disease (pages 16–17). Table 7 of the guideline summarizes possible applications of various imaging techniques in the diagnosis of heart failure. Thereby, the value of CMR is rated - on a scale from “+” to “+++” - with “+++” regarding coronary artery disease, myocarditis, sarcoidosis, amyloidosis, eosinophilic syndromes, iron overload, arrhythmogenic right ventricular cardiomyopathy, endomyocardial fibrosis; with “++” regarding valvular regurgitation, HCM, pericarditis, Takotsubo-cardiomyopathy, and with “+” regarding valvular stenosis and Anderson-Fabry-Disease. In patients presenting with heart failure and ECG signs of LV hypertrophy or low QRS voltage, CMR is recommended for further work-up. Table 15 provides recommendations for CMR in ambulatory patients suspected of having heart failure.

**Table 16:** Recommendations for CMR during pregnancy

Recommendations	Class^a^	Level^b^	Page
CMR (without gadolinium) should be considered if echocardiography is insufficient for diagnosis.	IIa	C	14
Imaging of the entire aorta (CT/CMR) should be performed before pregnancy in patients with Marfan syndrome or other known aortic disease.	I	C	22
For imaging of pregnant women with dilatation of the distal ascending aorta, aortic arch or descending aorta, CMR (without gadolinium) is recommended.	I	C	22

^a^ Class of recommendation

^b^ Level of evidence

### 2012 European guidelines on cardiovascular disease prevention in clinical practice [28]

This guideline dedicates a chapter to the early detection of cardiovascular disease in asymptomatic subjects by CMR. It concludes that at present, CMR is a promising research tool, but its routine use remains limited and it is not yet appropriate for identifying patients at high risk for cardiovascular disease (page 23).

### 2011 ESC/EAS guidelines for the management of dyslipidaemias [29]

This guideline does not contain relevant paragraphs regarding CMR.

### 2011 ESC guidelines on the management of cardiovascular diseases during pregnancy [30]

CMR may be useful in diagnosing complex heart disease or pathology of the aorta. Limited data during organogenesis are available, but CMR is probably safe, especially after the first trimester. Gadolinium can be assumed to cross the fetal blood-placental barrier, but data are limited. The long-term risks of exposure of the developing fetus to free gadolinium ions are not known, and therefore gadolinium should be avoided (page 8). In bicuspid aortic valve disease, dilatation is often maximal in the distal part of the ascending aorta, which cannot be adequately visualized echocardiographically; therefore, CMR or CT should be performed before pre-pregnancy (page 21). Table 16 summarizes recommendations for CMR during pregnancy.

**Table 17:** Recommendations for MRA to assess peripheral artery disease

Recommendations for evaluation of carotid artery stenosis	Class^a^	Level^b^	Page
Duplex ultrasound, CT-angiography, and/or MRA are indicated to evaluate carotid artery stenosis.	I	A	11
Recommendations for diagnosis of symptomatic chronic mesenteric ischaemia	Class^a^	Level^b^	Page
When Duplex ultrasound is inconclusive, CT-angiography or gadolinium-enhanced MRA are indicated.	I	B	19
Recommendations for diagnostic strategies for renal artery stenosis	Class^a^	Level^b^	Page
MRA (in patients with creatinine clearance >30 mL/min) is recommended to establish the diagnosis of renal artery stenosis.	I	B	21
Recommendations for diagnostic tests in patients with lower extremity artery disease	Class^a^	Level^b^	Page
Duplex ultrasound and/or CT-angiography and/or MRA are indicated to localize lower extremity artery disease lesions and consider revascularization options.	I	A	26

^a^ Class of recommendation

^b^ Level of evidence

### 2011 ESC guidelines on the diagnosis and treatment of peripheral artery diseases [31]

MR angiography (MRA) is regarded as one of the main diagnostic modalities to assess peripheral artery disease. Table 17 summarizes the recommendations for MRA to assess peripheral artery disease.

### 2010 ESC guidelines for the management of grown-up congenital heart disease [32]

CMR has become increasingly important in grown-up with congenital heart disease (GUCH) and is an essential facility in the specialist unit. ESC recommendations for the use of CMR in GUCH patients have been published separately [33]. There are several groups of indications for CMR when assessing adult congenital heart disease in clinical practice:

1. CMR as an alternative to echocardiography, when both techniques can provide similar information but echocardiography cannot be obtained with sufficient quality.
2. CMR as a second method when echocardiography measurements are borderline or ambiguous.
3. Indications where CMR is considered superior to echocardiography and should be regularly used when the information is essential for patient management. These indications include the quantification of right ventricular (RV) volumes and ejection fraction, RV and LV mass, evaluation of RV outflow tract and conduits, quantification of pulmonary regurgitation, evaluation of pulmonary arteries, aorta, systemic and pulmonary veins, collaterals and arteriovenous malformations, coronary anomalies and coronary artery disease, evaluation of intra- and extracardiac masses, and myocardial tissue characterization (fibrosis, fat, iron).

### 2010 focused update of ESC Guidelines on device therapy in heart failure [34]

CMR is not mentioned in this guideline.

### 2009 guidelines for the diagnosis and management of syncope [35]

In the diagnostic work-up of syncope, CMR - along with other imaging modalities - may be performed in selected cases (e.g. aortic dissection and haematoma, pulmonary embolism, cardiac masses, pericardial and myocardial diseases, congenital anomalies of coronary arteries) (page 23).

## Discussion

This is the first systematic summary of the representation of CMR in the ESC guidelines. It shows that CMR is mentioned in the majority of guidelines (89 %) and that more than 50 % of the guidelines contain specific recommendations, when and how to use CMR. Almost all recommendations are in favour of the use of CMR.

The majority of recommendations refer to stress imaging to assess coronary artery disease in general. Even though CMR is not listed as the only recommended modality, it is ranked equally to nuclear studies and stress-echocardiography. Recently, large and important studies like CE-Marc have promoted this favourable position of CMR [4]. Accordingly, the evaluation of suspected coronary artery disease or ischemia in known coronary artery disease makes up the largest indication group for CMR in the EuroCMR registry [2].

Interestingly, in the context of ischemic heart disease, the ESC guidelines are relatively conservative in the evaluation of CMR viability testing. They rate its ability to detect viability and predict recovery of wall motion no better than with other imaging techniques and do not word a specific recommendation [16]. By way of contrast, viability testing makes up the third largest indication group in the EuroCMR registry [2].

The use of CMR in HCM is also well represented in the corresponding guideline. Thereby, CMR is mainly recommended to describe the phenotype and make the diagnosis, while its value for risk stratification for sudden cardiac death is still under debate [36]. Other cardiomyopathies (e.g. DCM, ARVC) and myocarditis are less well expressed in specific ESC recommendations. This can be attributed to the lack of large-scale data, as well as the absence of specific ESC-guidelines dedicated to cardiomyopathies (other than HCM) or inflammatory heart disease. The significance of CMR in these indication groups is underlined by several ESC position statements: A recent document about myocarditis stated that CMR may be considered in clinically stable patients with myocarditis [37]. A recent document about cardiomyopathies stated that the incremental contribution of CMR to the diagnosis of cardiomyopathies derives from accurate assessment of the morphology and function of the heart and tissue characterization [38]. Finally, a document about the role of endomyocardial biopsy in the management of cardiovascular disease mentions CMR repeatedly as a valuable tool in patients scheduled for biopsy either to assist or to replace biopsy [39]. In those centers taking part in the EuroCMR registry, cardiomyopathies and myocarditis make up the second largest CMR indication group [2].

Other well-established indications for CMR are completely unmentioned in the ESC guidelines, like CMR in the context of sarcoid disease. A recent consensus statement by the Heart Rhythm Society from 2014 on the diagnosis and management of arrhythmias associated with cardiac sarcoid defined the presence of LGE on CMR as one criteria for the diagnosis of cardiac sarcoid [40]. Screening for cardiac involvement in patients with biopsy-proven extracardiac sarcoidosis should include advanced cardiac imaging like CMR under certain circumstances. Planning the ablation procedure based on the predominant location of scarring as detected by LGE-CMR may be helpful and CMR may support sudden death risk stratification. Nevertheless, for the present analysis we decided to stick only to the ESC guidelines to warrant a consistent level of guideline standard.

Regarding valvular and congenital heart disease, the ESC guidelines contain extensive text passages about the value of CMR, reflecting current practice, where these indications make up a substantial part of all examinations [2]. In future guideline versions, the translation of these paragraphs into specific recommendations is needed to clarify the position of CMR.

This study touches several aspects: First, the frequent representation of CMR in the ESC guidelines demonstrates that the cardiology society has accepted CMR as an integral part of the armamentarium of cardiovascular diagnostic modalities (e.g. stress testing). As a next step, studies are needed that analyse the adherence to the ESC guidelines and how it impacts patients’ management [41]. Second, there are several clinical scenarios, where CMR is already used at dedicated centres, but which are not well represented in the ESC guidelines (e.g. myocarditis). Here, further studies are needed to provide the required evidence. Third, CMR has not yet arrived in the clinical reality in many regions of Europe. Hence, not everywhere in Europe can the patients be managed according to the ESC guidelines. The reasons are certainly multifactorial, with economic issues playing a central role: i) in some diseases alternative techniques are often readily available that provide similar information as CMR does. This is especially true for testing for myocardial ischemia, where SPECT and stress echocardiography are still the dominant modalities. ii) CMR is recognized as expensive and reimbursement not aspired by the medical insurances in many countries. iii) knowledge both to run a CMR examination and to interpret the images with profound cardiologic knowledge is often limited and structures for systematic training are needed, including the establishment of cooperation between radiologists and cardiologists.

Already now, there are attempts how to overcome the latter obstacles and to enable the use of CMR in accordance with the guidelines: Recent large-scale studies demonstrated the diagnostic accuracy of CMR and its superiority in some indications [4]; prognostic data are available that demonstrate the benefit of CMR [42]; there are studies that demonstrate the potential for saving resources by using CMR [43]; structures for acquiring CMR skills including e-based learning are evolving [44]; and CMR imaging became faster and the user interfaces easier to handle.

### Limitations of the study

This summary is not intended to provide a balanced comparison of the various imaging modalities in the ESC guidelines, but aimed at describing only the role of CMR.

## Conclusions

CMR is represented in the majority of the ESC guidelines. They contain many recommendations in favour of the use of CMR in specific scenarios. Issues regarding training, costs and reimbursement have to be solved to provide CMR to the patients in accordance with the ESC recommendations.

## Appendix: Summary of clinical scenarios/diagnoses, where the ESC made recommendations regarding CMR

**Table:**

Suspected/stable coronary artery disease	Class^a^	Level^b^	Guideline
Whenever history suggests myocardial ischaemia, a stress ECG test is recommended, and, if positive or ambiguous, an imaging stress test (stress echocardiography, stress CMR or nuclear scintigraphy) is recommended.	I	C	[22]
In subjects with intermediate pretest probability for suspected coronary artery disease and stable symptoms, stress CMR, stress-echo, SPECT or PET are recommended	I	A	[16]
In patients with suspected stable coronary artery disease and intermediate pretest probability of 15 % - 65 % and LVEF =50 %, stress imaging is preferred as the initial test option if local expertise and availability permit.	I	B	[20]
An imaging stress test is recommended as the initial test for diagnosing stable coronary artery disease if the pretest probability is between 66-85 % or if LVEF is <50 % in patients without typical angina.	I	B	[20]
An imaging stress test is recommended in patients with resting ECG abnormalities, which prevent accurate interpretation of ECG changes during stress.	I	B	[20]
Stress imaging for risk stratification is recommended in patients with a non-conclusive exercise ECG	I	B	[20]
Risk stratification is recommended based on clinical assessment and the results of the stress test initially employed for making a diagnosis of stable coronary artery disease	I	B	[20]
In asymptomatic adults with diabetes or asymptomatic adults with a strong family history of coronary artery disease or when previous risk assessment testing suggests high risk of coronary artery disease, such as a coronary artery calcium score of 400 or greater stress imaging tests (MPI, stress echocardiography, perfusion CMR) may be considered for advanced cardiovascular risk assessment.	IIb	C	[20]
In patients with stable coronary disease after a significant change in symptom level, risk stratification using stress ECG (unless they cannot exercise or display ECG changes which make the ECG non evaluable) or preferably stress imaging if local expertise and availability permit is recommended	I	B	[20]
In patients with known stable coronary artery disease and a deterioration in symptoms, stress imaging is recommended for risk stratification if the site and extent of ischemia would influence clinical decision making	I	B	[20]
An exercise ECG or stress imaging if appropriate is recommended in the presence of recurrent or new symptoms once instability has been ruled out.	I	C	[20]
Reassessment of the prognosis using stress testing may be considered in asymptomatic patients after the expiration of the period for which the previous test was felt to be valid (“warranty period”)	IIb	C	[20]
Risk stratification before non-cardiac surgery	Class^a^	Level^b^	Guideline
Imaging stress testing is recommended before high-risk surgery in patients with more than two clinical risk factors and poor functional capacity (<4 METs).	I	C	[18]
Imaging stress testing may be considered before high- or intermediate-risk surgery in patients with one or two clinical risk factors and poor functional capacity (<4 METs).c	IIb	C	[18]
Imaging stress testing is not recommended before low-risk surgery, regardless of the patient’s clinical risk.	III	C	[18]
Acute coronary syndrome	Class^a^	Level^b^	Guideline
In patients with no recurrence of chest pain, normal ECG findings and normal levels of cardiac troponin (preferably high-sensitivity), but suspected acute coronary syndrome, a non-invasive stress test (preferably with imaging) for inducible ischaemia is recommended before deciding on an invasive strategy.	I	A	[9]
If echocardiography is not feasible, CMR may be used as an alternative for assessment of infarct size and resting LV function after STEMI.	IIb	C	[26]
For patients with multivessel disease, or in whom revascularization of other vessels is considered, stress testing or imaging (e.g. using stress myocardial perfusion scintigraphy, stress echocardiography, positron emission tomography or CMR) for ischaemia and viability is indicated after STEMI before or after discharge.	I	A	[26]
Before coronary revascularization	Class^a^	Level^b^	Guideline
An imaging stress test should be considered to assess the functional severity of intermediate lesions on coronary arteriography.	IIa	B	[20]
To achieve a prognostic benefit by revascularization in patients with coronary artery disease, ischemia has to be documented by non-invasive imaging	I	A-C	[16]
After coronary revascularization	Class^a^	Level^b^	Guideline
In asymptomatic patients after revascularisation, early imaging testing should be considered in specific patient subsets.	IIa	C	[16]
Late (6 months) stress imaging test after revascularization may be considered to detect patients with restenosis after stenting or graft occlusion irrespective of symptoms.	IIb	C	[20]
In asymptomatic patients, routine stress testing may be considered >2 years after PCI and >5 years after CABG.	IIa	B	[16]
In symptomatic patients with revascularized stable coronary artery disease, stress imaging (stress echocardiography, CMR or MPS) is indicated rather than stress ECG.	I	C	[20]
In symptomatic patients with prior revascularization (PCI or CABG), an imaging stress test should be considered	IIa	B	[20]
In symptomatic patients after revascularization with low-risk findings at stress testing, it is recommended to reinforce medical therapy and lifestyle changes.	I	C	[16]
In symptomatic patients after revascularization with intermediate- to high-risk findings at stress testing, coronary angiography is recommended.	I	C	[16]
Heart failure	Class^a^	Level^b^	Guideline
CMR imaging is recommended to evaluate cardiac structure and function, to measure LVEF, and to characterize cardiac tissue, especially in subjects with inadequate echocardiographic images or where the echocardiographic findings are inconclusive or incomplete (but taking account of cautions/contraindications to CMR).	I	C	[27]
Myocardial perfusion/ischaemia imaging (echocardiography, CMR, SPECT, or PET) should be considered in patients thought to have coronary artery disease, and who are considered suitable for coronary revascularization, to determine whether there is reversible myocardial ischaemia and viable myocardium.	IIa	C	[27]
Ventricular arrhythmia	Class^a^	Level^b^	Guideline
Pharmacological stress testing plus imaging modality is recommended to detect silent ischaemia in patients with ventricular arrhythmias who have an intermediate probability of having coronary artery disease by age or symptoms and are physically unable to perform a symptom-limited exercise test.	I	B	[6]
CMR should be considered in patients with ventricular arrhythmias when echocardiography does not provide accurate assessment of LV and RV function and/or evaluation of structural changes.	IIa	B	[6]
Inflammatory heart disease	Class^a^	Level^b^	Guideline
Demonstration of persistent myocardial inflammatory infiltrates by immunohistological evidence and/or abnormal localized fibrosis by CMR after acute myocarditis may be considered as an additional indicator of increased risk of SCD in inflammatory heart disease.	IIb	C	[6]
CMR is recommended for the confirmation of myocardial involvement in pericarditis	I	C	[11]
Hypertrophic cardiomyopathy	Class^a^	Level^b^	Guideline
It is recommended that CMR studies in suspected HCM be performed and interpreted by teams experienced in cardiac imaging and in the evaluation of heart muscle disease	I	B	[14]
In the absence of contraindications, CMR with LGE is recommended in patients with suspected HCM who have inadequate echocardiographic windows, in order to confirm the diagnosis.	I	C	[14]
In the absence of contraindications, CMR with LGE should be considered in patients fulfilling diagnostic criteria for HCM, to assess cardiac anatomy, ventricular function, and the presence and extent of myocardial fibrosis.	IIa	B	[14]
CMR with LGE imaging should be considered in patients with suspected apical hypertrophy or aneurysm.	IIa	C	[14]
CMR with LGE may be considered before septal alcohol ablation or myectomy, to assess the extent and distribution of hypertrophy and myocardial fibrosis.	IIb	C	[14]
CMR may be considered every 5 years in clinically stable patients, or every 2–3 years in patients with progressive disease.	IIb	C	[14]
Athlete’s heart	Class^a^	Level^b^	Guideline
For prevention of sudden cardiac death in athletes, upon identification of ECG abnormalities suggestive of structural heart disease, echocardiography and/or CMR imaging is recommended.	I	C	[6]
Storage disease	Class^a^	Level^b^	Guideline
CMR with LGE imaging should be considered in patients with suspected cardiac amyloidosis.	IIa	C	[14]
Pericardial diseases	Class^a^	Level^b^	Guideline
CMR is second-level testing for diagnostic workup in pericarditis	I	C	[11]
CMR should be considered in suspected cases of loculated pericardial effusion, pericardial thickening and masses, as well as associated chest abnormalities	IIa	C	[11]
CMR is indicated as second-level imaging technique to assess pericardial thickness, degree and extension of pericardial involvement for the diagnosis of constrictive pericarditis	I	C	[11]
Empiric anti-inflammatory therapy may be considered in cases with transient or new diagnosis of constrictive pericarditis with concomitant evidence of pericardial inflammation (i.e. pericardial enhancement on CMR)	IIb	C	[11]
Pregnancy	Class^a^	Level^b^	Guideline
CMR (without gadolinium) should be considered if echocardiography is insufficient for diagnosis.	IIa	C	[30]
Imaging of the entire aorta (CT/CMR) should be performed before pregnancy in patients with Marfan syndrome or other known aortic disease.	I	C	[30]
For imaging of pregnant women with dilatation of the distal ascending aorta, aortic arch or descending aorta, CMR (without gadolinium) is recommended.	I	C	[30]
Vessel disease	Class^a^	Level^b^	Guideline
In stable patients with a suspicion of acute aortic syndrome, CMR is recommended (or should be considered) according to local availability and expertise	I	C	[15]
In case of initially negative imaging with persistence of suspicion of acute aortic syndrome, repetitive imaging (CT or CMR) is recommended.	I	C	[15]
In case of uncomplicated Type B aortic dissection treated medically, repeated imaging (CT or CMR) during the first days is recommended.	I	C	[15]
In uncomplicated Type B intramural hematoma, repetitive imaging (CMR or CT) is indicated.	I	C	[15]
In uncomplicated Type B penetrating aortic ulcer, repetitive imaging (CMR or CT) is indicated.	I	C	[15]
CMR or CT is indicated in patients with bicuspid aortic valve when the morphology of the aortic root and the ascending aorta cannot be accurately assessed by TTE.	I	C	[15]
In the case of aortic diameter >50 mm or an increase >3 mm/year measured by echocardiography, confirmation of the measurement is indicated, using another imaging modality (CT or CMR).	I	C	[15]
Contrast CT or CMR is recommended to confirm the diagnosis of chronic aortic dissection.	I	C	[15]
For follow-up after (T)EVAR in young patients, CMR should be preferred to CT for magnetic resonance-compatible stent grafts, to reduce radiation exposure.	IIa	C	[15]
CMR, CT, or digital subtraction angiography may be considered if carotid artery stenosis by ultrasound is >70 % and myocardial revascularization is contemplated.	IIb	C	[16]
MR angiography should not be used to rule out pulmonary embolism.	III	C	[17]
Duplex ultrasound, CT-angiography, and/or MRA are indicated to evaluate carotid artery stenosis.	I	A	[31]
When Duplex ultrasound is inconclusive, CT-angiography or gadolinium-enhanced MRA are indicated to evaluate chronic mesenteric ischaemia.	I	B	[31]
MRA (in patients with creatinine clearance >30 mL/min) is recommended to establish the diagnosis of renal artery stenosis.	I	B	[31]
Duplex ultrasound and/or CT-angiography and/or MRA are indicated to localize lower extremity artery disease lesions and consider revascularization options.	I	A	[31]

^a^ Class of recommendation

^b^ Level of evidence

## Abbreviations

CMR: cardiovascular magnetic resonance
CT: computed tomography
ECG: electrocardiography
ESC: European society for cardiology
LAD: left anterior descending coronary artery
LGE: late gadolinium enhancement
LV: left ventricle
MRA: magnetic resonance angiography
PET: positron emission tomography
RV: right ventricle
SPECT: single photon emission computed tomography
STEMI: ST elevation myocardial infarct
TTE: transthoracic echocardiography
